# Sex Differences in Overall Survival Among Patients with Non-Small-Cell Lung Cancer Across Clinical Stages: A Population-Based SEER Study

**DOI:** 10.3390/healthcare14070966

**Published:** 2026-04-07

**Authors:** Yuan Li, Takayuki Noma, Long Liang, Wenhao Weng

**Affiliations:** 1 Center for Clinical Research and Translational Medicine, Yangpu Hospital, Tongji University School of Medicine, Tongji University, Shanghai 200070, China; maxly0828@163.com; 2 Department of Molecular Diagnostics and Experimental Therapeutics, Beckman Research Institute of City of Hope, Biomedical Research Center, Monrovia, CA 91016, USA; tnoma@coh.org; 3 Department of Respiratory and Critical Care Medicine, Health Science Center, Peking University Third Hospital, Peking University, Beijing 100191, China; 4 School of Medicine, Shanghai Children’s Hospital, Shanghai Jiao Tong University, Shanghai 200062, China

**Keywords:** sex, non-small-cell lung carcinoma, overall survival (OS), chemotherapy, multivariable cox analysis

## Abstract

Background/Objective: Sex-based disparities in cancer outcomes have gained increasing attention in women’s health research. We examined the relationship between sex and overall survival (OS) among patients with non-small-cell lung cancer (NSCLC), with particular emphasis on the survival advantage observed in women across different clinical stages and treatment settings. Sex-related differences in cancer outcomes have become an important focus in oncology and women’s health research. This study aimed to investigate the association between sex and overall survival (OS) in patients with non-small-cell lung cancer (NSCLC), with particular attention to the observed survival advantage in women across clinical stages and treatment contexts. Methods: A total of 129,864 patients diagnosed with NSCLC were identified, including 78,460 men and 51,404 women. Demographic characteristics, socioeconomic status, tumor features, treatment modalities, and survival outcomes were compared between sexes. Kaplan–Meier survival analyses and stage-stratified Cox proportional hazards models were used to evaluate overall survival differences between female and male patients. Results: Women demonstrated significantly superior OS compared with men across all stages of NSCLC (all *p* < 0.001). This survival advantage persisted regardless of receipt of chemotherapy. Among patients receiving chemotherapy, survival improvements were observed in both sexes; however, women consistently exhibited longer median OS at each stage. From stage IB to IV, median OS in women was 52.0, 30.0, 13.0, and 5.0 months, respectively, compared with 33.0, 23.0, 11.0, and 4.0 months in men. Notably, the magnitude of sex-related survival differences was more pronounced in earlier stages (IB/II) than in advanced stages (III/IV), suggesting potential biological or treatment response differences favoring women. Age-stratified analyses further demonstrated that women older than 45 years experienced a consistent survival advantage across all stages. Multivariable Cox regression confirmed that female sex was independently associated with reduced mortality risk at every stage (HRs ranging from 0.766 to 0.857; all *p* < 0.001). Conclusions: Women with NSCLC exhibit a significant and independent survival advantage over men across clinical stages, regardless of chemotherapy status, particularly among patients older than 45 years. These findings highlight the importance of considering sex in prognostic assessment and support further investigation into factors contributing to survival differences in NSCLC.

## 1. Introduction

Lung cancer is one of the most diagnosed malignancies and remains the leading cause of cancer-related mortality worldwide [1]. Although historically considered a predominantly male disease, the incidence of lung cancer among women has risen substantially over the past decades, making it an increasingly important global women’s health concern. Global estimates indicate that lung cancer accounts for approximately 1.7 million deaths each year [2]. Lung cancer is broadly classified into non-small-cell lung cancer (NSCLC) and small-cell lung cancer (SCLC), with NSCLC accounting for approximately 85% of all cases. The major histological subtypes of NSCLC comprise squamous-cell carcinoma (SCC), adenocarcinoma (AD), and large-cell neuroendocrine carcinoma (LCNC).

Treatment strategies for NSCLC are largely determined by tumor stage. Surgical resection remains the standard and most appropriate treatment for patients with early-stage disease. However, postoperative survival outcomes vary considerably and depend on patients’ physical status, tumor stage, and histological subtype. Previous studies have reported the 5-year survival following surgical resection for NSCLC remains around 25% [2,3,4,5]. Moreover, the risk of recurrence persists even after complete resection.

To improve long-term outcomes, chemotherapy and/or radiotherapy are frequently administered following surgical resection to eradicate residual malignant cells and limit metastatic spread. The beneficial effect of chemotherapy on overall survival (OS) has been consistently demonstrated across multiple clinical trials [6,7]. Furthermore, studies have demonstrated that chemotherapy has been linked to improved survival outcomes in patients with NSCLC, including elderly individuals and those with comorbidities [8,9,10].

Emerging evidence suggests that lung cancer in women may differ from that in men in terms of epidemiological characteristics, tumor biology, treatment response, and survival outcomes [11,12]. Several studies have reported improved survival among women with NSCLC; however, the magnitude of this advantage across different clinical stages remains incompletely understood [13]. In addition, whether sex influences the survival benefit derived from chemotherapy has not been comprehensively evaluated.

Understanding sex-specific survival patterns is essential for optimizing individualized treatment strategies and advancing precision medicine in women’s health.

Therefore, this population-based cohort study was conducted to assess whether sex influences overall survival in patients with NSCLC treated with and without chemotherapy across clinical stages IB–IV.

## 2. Methods

### 2.1. Data Acquisition and Cohort Definition

This population-based study utilized data from the Surveillance, Epidemiology, and End Results (SEER) database, a nationwide program consisting of 18 cancer registries in the United States [14]. The SEER database covers approximately 28% of the U.S. population and provides comprehensive clinical and demographic information for cancer patients [15]. It includes variables related to socio-demographic factors (e.g., geographic region, education level, and household income) as well as tumor characteristics such as histology, size, and stage. These data enable large-scale analyses of treatment outcomes and survival.

Patients diagnosed with non-small-cell lung cancer (NSCLC) between 2004 and 2015 were identified from the SEER database. Tumor staging was determined using the Derived AJCC Stage Group variable, which integrates staging information across different AJCC editions and allows for consistent classification across calendar years. Based on this variable, patients were grouped into stages IB, II, III, and IV for subsequent analyses. Patients were excluded if they had incomplete or missing data on survival or tumor stage, were lost to follow-up, or had lung cancer that was not their first primary malignancy.

**Figure 1:**
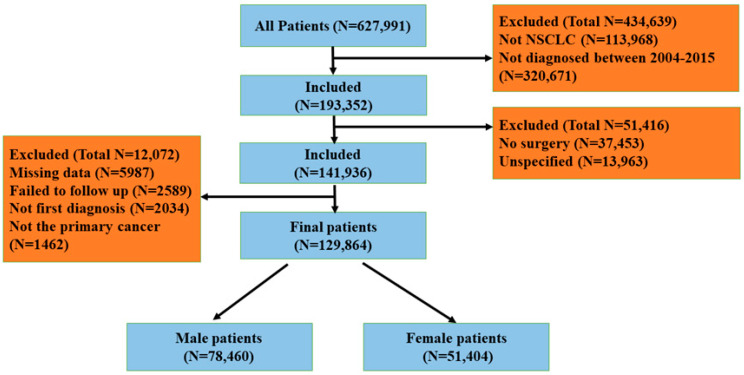
Patient selection flowchart. Flowchart of patient selection from the SEER database (2004–2015). After applying inclusion and exclusion criteria, a total of 129,864 patients with NSCLC were included in the final analysis, comprising 78,460 men and 51,404 women.

In total, 129,864 patients with pathologically confirmed NSCLC were included in the final analysis, comprising 78,460 males and 51,404 females. All included patients underwent either surgical treatment alone or surgery combined with chemotherapy. The patient selection process is illustrated in Figure 1.

### 2.2. Study Variables

Clinical and demographic data of patients with NSCLC were obtained from the SEER database. The variables extracted included race, sex, geographic region, primary tumor location, tumor grade, laterality, histological subtype, number of lymph nodes examined, tumor stage, chemotherapy status, and the presence of bone, brain, liver, or lung metastases. Additional variables included first malignant indicator, age at diagnosis, insurance status, marital status, level of high school education, and median family income.

Chemotherapy was defined as a binary variable (yes vs. no) according to SEER registry coding. Detailed information regarding chemotherapy regimen, administration route, timing (adjuvant, neoadjuvant, or palliative), dose intensity, and treatment intent is not available in the SEER database.

Overall, 20 variables were incorporated into the subsequent univariate and multivariable statistical analyses.

### 2.3. Statistical Methods and Analysis

All analyses were conducted using IBM SPSS software (version 20.0; IBM Corp., Armonk, NY, USA). Baseline characteristics between male and female patients were compared using the chi-square test for categorical variables. Overall survival (OS) was estimated using the Kaplan–Meier method, and differences between groups were evaluated with the log-rank test. Median follow-up time was calculated using the reverse Kaplan–Meier approach. To account for potential confounding, multivariable Cox proportional hazards models were constructed to identify independent predictors of OS for each clinical stage. The proportional hazards assumption was assessed during model diagnostics, and no significant violations were observed for the primary exposure variable (sex) in the stage-specific models. A two-sided *p*-value < 0.05 was considered statistically significant.

## 3. Results

### 3.1. Characteristics of the Study Population

A total of 129,864 patients with NSCLC were included in this population-based study, comprising 78,460 men and 51,404 women. Baseline demographic, clinicopathological, and socioeconomic characteristics stratified by sex are summarized in Table 1.

In both groups, the majority of patients were White (men: 81.8%; women: 83.3%) and predominantly located in the eastern region (49.5% vs. 46.6%). Tumors were most frequently found in the upper lung lobe (52.1% vs. 50.7%) and exhibited a right-sided predominance (55.2% vs. 56.0%). A substantial proportion of tumors were poor differentiation (Grade III/IV) was observed in a considerable proportion of cases (34.0% in men vs. 32.4% in women). Notably, histological distributions differed by sex. Squamous-cell carcinoma was more common in men (78.7% vs. 66.9%), whereas adenocarcinoma was more frequently observed in women (24.5% vs. 13.3%). The proportion of large-cell carcinoma was comparable between groups (8.0% vs. 8.6%). Most patients presented with advanced-stage disease (stage III/IV: 73.9% in men vs. 71.0% in women), and nearly half of patients received chemotherapy (47.5% vs. 44.7%). Rates of distant metastases were relatively low in both groups, including bone (7.3% vs. 6.0%), brain (3.6% vs. 3.8%), liver (3.9% vs. 3.2%), and lung metastases (6.8% vs. 6.7%). Most patients were elderly (age ≥ 65 years: 68.5% vs. 71.3%) and had a positive first malignant indicator (77.0% vs. 77.2%). Regarding socioeconomic characteristics, the majority had insurance coverage (73.0% vs. 74.1%), higher educational attainment (80.2% vs. 79.0%), and higher household income levels (85.7% vs. 88.7%). Differences in marital status were also observed between sexes.

Overall, these findings indicate distinct clinicopathological and demographic patterns between male and female patients with NSCLC.

### 3.2. Kaplan–Meier OS and Median Survival for NSCLC Patients Stratified by Sex and Age at Stages IB Through IV

**Figure 2:**
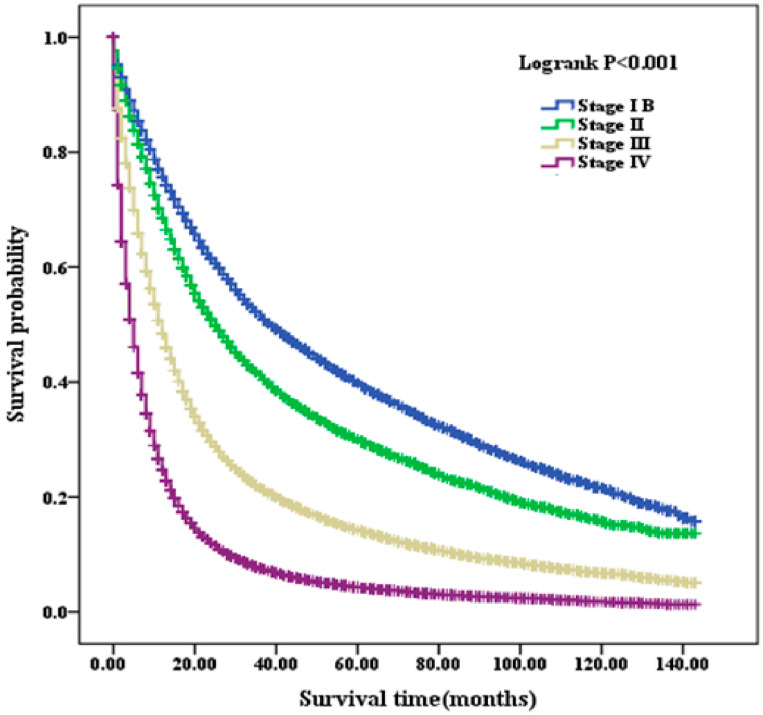
Overall survival across clinical stages IB–IV in patients with NSCLC. NSCLC, non-small-cell lung cancer.

**Figure 3:**
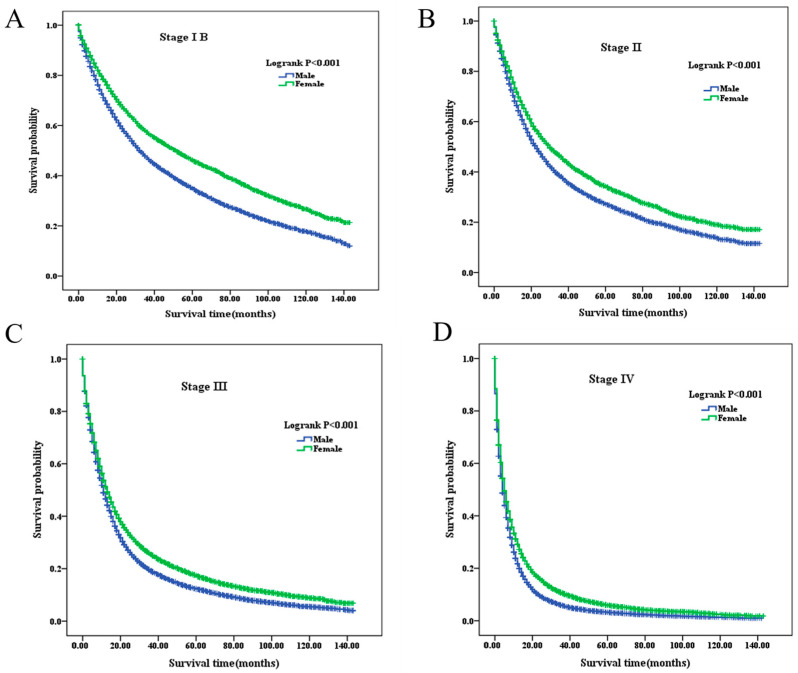
Overall survival comparison in male and female NSCLC patients across clinical stages IB–IV. (**A**): Stage IB; (**B**): Stage II; (**C**): Stage III; (**D**): Stage IV. NSCLC, non-small-cell lung cancer.

The overall survival (OS) for the entire cohort was 21.8%. Stratified by stage, the OS rates were 42.6%, 34.2%, 20.8%, and 10.0% for stages IB, II, III, and IV, respectively (Figure 2). Stage-specific survival analysis demonstrated that female patients consistently had better survival than male patients across all stages (log-rank test, all *p* < 0.001) (Figure 3). Kaplan–Meier curves consistently showed improved survival among women throughout stages IB to IV (*p* < 0.001). Further stratification by age revealed similar survival trends across most subgroups (*p* < 0.005), with female patients demonstrating superior survival. However, among younger patients (<45 years) in stages IB and II, the survival differences between sexes did not reach statistical significance (*p* = 0.055 and *p* = 0.096, respectively) (Figures S1–S4). The mean survival times for male patients at stages IB through IV were 52.82, 44.19, 25.09, and 10.78 months, respectively. In comparison, female patients exhibited longer mean survival times of 66.29, 52.87, 31.35, and 15.14 months (Table 2). Similarly, median OS decreased from 33.00 to 4.00 months across stages IB–IV in male patients and from 52.00 to 5.00 months in female patients across stages IB–IV. Detailed estimates with corresponding 95% confidence intervals are provided in Table 2.

### 3.3. Chemotherapy and Survival Outcomes Stratified by Sex Across Stages IB–IV

**Figure 4:**
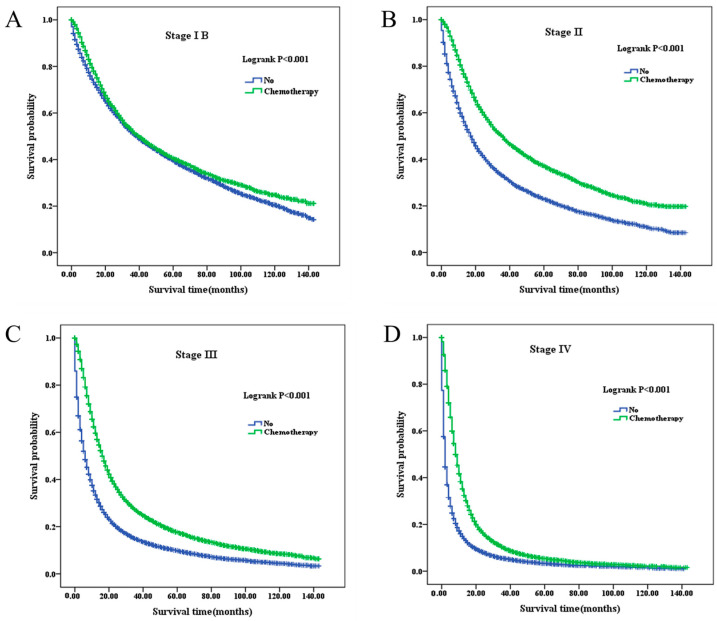
Overall survival according to chemotherapy status in patients with NSCLC across stages IB–IV. (**A**): Stage IB; (**B**): Stage II; (**C**): Stage III; (**D**): Stage IV. NSCLC, non-small-cell lung cancer.

**Figure 5:**
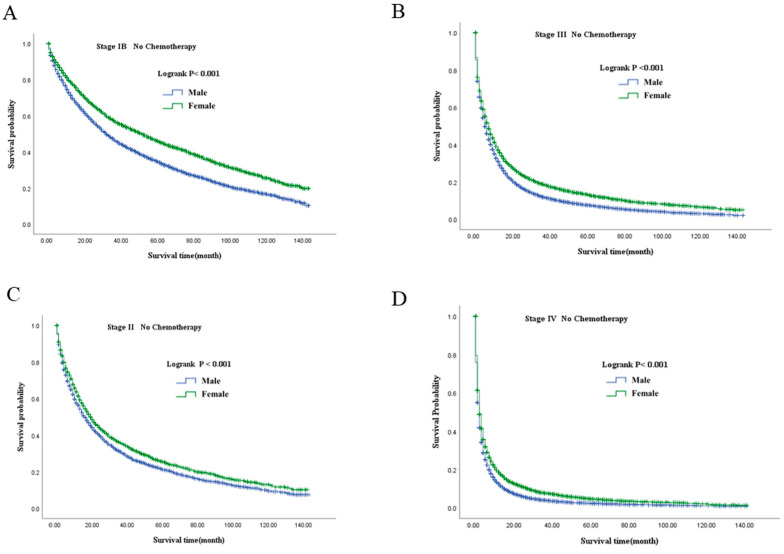
Overall survival stratified by sex in patients with NSCLC without chemotherapy across stages IB–IV. (**A**): Stage IB; (**B**): Stage III; (**C**): Stage II; (**D**): Stage IV. NSCLC, non-small-cell lung cancer.

**Figure 6:**
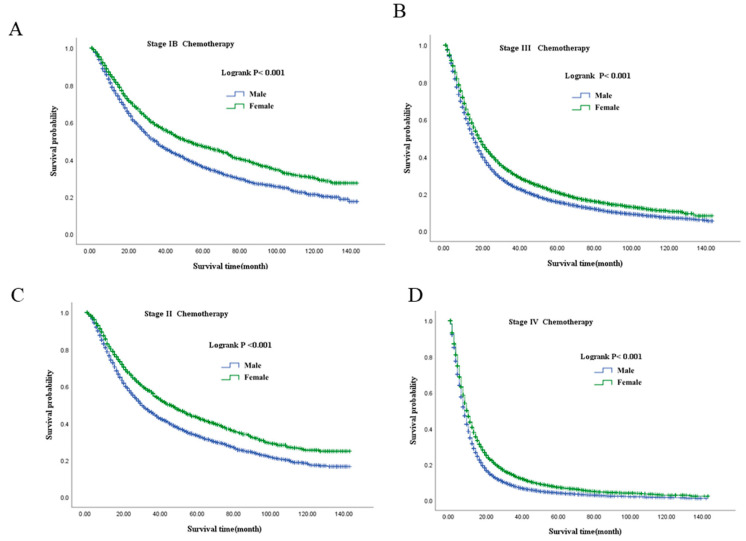
Overall survival stratified by sex in patients with NSCLC with chemotherapy across stages IB–IV. (**A**): Stage IB; (**B**): Stage III; (**C**): Stage II; (**D**): Stage IV. NSCLC, non-small-cell lung cancer.

Kaplan–Meier analyses demonstrated consistent sex-based differences in overall survival (OS) across all clinical stages, regardless of chemotherapy status (Figure 4, Figure 5 and Figure 6). In both chemotherapy-treated and untreated groups, female patients consistently exhibited superior survival compared with male patients at each stage (all log-rank *p* < 0.001).

Among patients who did not receive chemotherapy, females showed prolonged survival relative to males across stages IB–IV, as illustrated in Figure 5. For example, in stage II disease, median OS was 19.00 months (95% CI: 17.34–20.66) in female patients compared with 16.00 months (95% CI: 14.87–17.13) in male patients. Similarly, in stage III, median OS was 7.00 months (95% CI: 6.61–7.39) in females versus 5.00 months (95% CI: 4.78–5.22) in males.

In the chemotherapy-treated cohort, female patients maintained a survival advantage over male patients at all stages (Figure 6). For instance, in stage II, median OS was 46.00 months (95% CI: 42.01–49.99) in females compared with 30.00 months (95% CI: 27.95–32.05) in males, while in stage IV, median OS was 10.00 months (95% CI: 9.73–10.28) versus 8.00 months (95% CI: 7.84–8.16), respectively.

These findings indicate that the observed survival benefit in female patients is robust and persists irrespective of treatment status. Notably, while chemotherapy was associated with improved survival in both sexes, the relative survival advantage of female patients remained evident within each treatment subgroup.

Overall, female patients exhibited longer median OS than male patients at corresponding stages, regardless of chemotherapy status (Table 3).

### 3.4. Multivariable Cox Regression Analysis

Multivariable Cox regression analysis demonstrated that several demographics, clinicopathological, and socioeconomic factors were independently associated with overall survival across clinical stages. Notably, female sex was consistently associated with a reduced risk of mortality at all stages, with hazard ratios ranging from 0.766 to 0.857 (all *p* < 0.001).

Advancing age was strongly associated with worse survival, with patients aged ≥75 years exhibiting the highest mortality risk across all stages. In addition, unfavorable tumor characteristics—including higher grade, non-adenocarcinoma histology, and specific primary tumor locations—were associated with poorer outcomes. Treatment-related variables also showed significant associations with survival. Receipt of chemotherapy and a greater number of lymph nodes removed were consistently linked to improved survival across stages. Conversely, the presence of distant metastases (including bone, brain, liver, and lung) was associated with markedly increased mortality risk in advanced-stage disease.

Socioeconomic factors, such as insurance status, marital status, and household income, were also independently associated with survival outcomes, suggesting that both clinical and non-clinical factors contribute to prognosis in patients with NSCLC (Table 4).

## 4. Discussion

In this large population-based cohort, most patients with NSCLC were elderly, had poorly differentiated tumors, underwent removal of 0–3 lymph nodes, and were diagnosed at advanced stages. Nearly half of the cohort received adjuvant chemotherapy, while the overall proportion of distant metastases remained relatively low.

This study provides a stage-specific assessment of the relationship between chemotherapy and survival outcomes in both male and female patients with NSCLC from stages IB to IV. Across all clinical stages, female patients consistently exhibited a lower risk of mortality compared with male patients. This pattern remained stable after stratification by age, suggesting that the observed difference cannot be fully attributed to age-related factors. When treated with chemotherapy, both male and female patients demonstrated improved survival compared with those who did not receive chemotherapy. These findings suggest that chemotherapy confers significant therapeutic benefits regardless of sex. However, women consistently exhibited longer overall and median survival at corresponding stages.

Although prior studies have reported sex-related survival differences in overall NSCLC populations [16,17,18,19] detailed stage-specific analyses remain limited. Our study addresses this gap by demonstrating that sex independently influences survival outcomes at each stage. Interestingly, no statistically significant survival advantage was observed among younger patients (<45 years) with early-stage disease (IB/II), suggesting that the magnitude of sex-based differences may vary according to age and disease stage. As expected, survival declined substantially with advancing stages in both sexes. For example, female patients in early-stage disease experienced a markedly greater median survival advantage compared with males, whereas this difference narrowed in advanced-stage disease. These findings suggest that sex-related biological or treatment-response factors may exert a stronger influence in earlier stages. Age was identified as an independent prognostic factor across all stages. Increasing age was associated with higher mortality risk, potentially reflecting a greater burden of comorbidities and poorer performance status in older patients [20,21,22]. Nevertheless, our findings are consistent with previous reports demonstrating that elderly patients can derive meaningful survival benefits from chemotherapy [23,24].

Using the SEER database, we further demonstrated that patients with stages IB through IV NSCLC benefit from chemotherapy, particularly those with advanced-stage disease. Survival among stage III/IV patients receiving chemotherapy was substantially longer compared with those who did not receive chemotherapy. These findings are consistent with previous clinical studies supporting the survival benefit of chemotherapy in NSCLC [25,26,27,28,29]. However, some studies have reported limited or stage-dependent benefit, particularly in early-stage disease [30,31], contributing to ongoing debate regarding optimal patient selection.

Importantly, multivariable Cox regression analyses confirmed that sex, age, tumor grade, histological subtype, lymph node status, chemotherapy, first malignant indicator, and socioeconomic variables were significant predictors of mortality. These findings underscore the multifactorial nature of survival outcomes in NSCLC. Although treatment guidelines are stage-based, individualized decision-making should also consider tumor biology, differentiation, patient age, and comorbid conditions rather than relying solely on stage.

The strengths of this study include its large sample size and multi-institutional representation, which enhance statistical power and generalizability compared with single-center studies. However, several limitations should be acknowledged. First, key clinical variables, including smoking history, comorbidity burden, and performance status, were not available in the SEER database, residual confounding cannot be excluded [32,33]. Second, chemotherapy information in the SEER database is recorded as a binary variable (yes vs. no) without detailed information regarding regimen, timing, or treatment intent. As a result, we were unable to distinguish adjuvant from palliative chemotherapy. The observed associations between chemotherapy and overall survival should thus be interpreted as reflecting receipt of chemotherapy rather than a specific treatment strategy. Third, the long study period (2004–2015), together with the lack of molecular and treatment data—such as driver mutations (e.g., EGFR, ALK, KRAS), PD-L1 expression, and the use of targeted or immunotherapy—may introduce heterogeneity in treatment patterns and influence survival outcomes [34]. However, in calendar period–stratified analyses, the association between chemotherapy and survival, as well as the observed sex-related survival difference, remained consistent across all time periods, suggesting that temporal changes in treatment practice are unlikely to have substantially affected the main findings of this study. Future studies incorporating detailed molecular profiling and treatment information are warranted to further elucidate these associations. Despite these limitations, the large sample size and population-based design provide robust evidence supporting a consistent survival difference between female and male patients with NSCLC across clinical stages.

## Figures and Tables

**Table 1:** Baseline Characteristics of the Study Population.

Covariate	Male (n, %)	Female (n, %)
Total	(78,460)	(51,404)
Race		
White	64,166 (81.8)	42,800 (83.3)
Black	9745 (12.4)	5994 (11.7)
Others	4549 (5.8)	2610 (5.1)
Region		
East	38,810 (49.5)	23,941 (46.6)
North	9101 (11.6)	5929 (11.5)
Southwest	2190 (2.8)	1346 (2.6)
Northwest	28,359 (36.1)	20,188 (39.3)
Primary Site		
Upper lobe	40,840 (52.1)	26,072 (50.7)
Middle lobe	2878 (3.7)	2067 (4.0%)
Lower lobe	21,843 (27.8)	15,500 (30.2)
NOS	6949 (8.9)	4709 (9.2)
Overlapping lesion	1156 (1.5)	695 (1.4)
Main bronchus	4794 (6.1)	2361 (4.6)
Grade		
Grade I	3288 (4.2)	3484 (6.8)
Grade II	19,250 (24.5)	12,522 (24.4)
Grade III	24,628 (31.4)	15,206 (29.6)
Grade IV	2055 (2.6)	1418 (2.8)
Unknow	29,239 (37.3)	18,774 (36.5)
Laterality		
Right	43,278 (55.2)	28,772 (56.0)
Left	32,745 (41.7)	21,010 (40.9)
Bilateral	909 (1.2)	663 (1.3)
Others	1528 (1.9)	959 (1.9)
Histological type		
SCC	61,754 (78.7)	34,406 (66.9)
AD	10,467 (13.3)	12,593 (24.5)
LCC	6239 (8.0)	4405 (8.6)
Lymph nodes removed		
0–3 lymph nodes	57,662 (73.5)	36,458 (70.9)
≥4 lymph nodes	15,303 (19.5)	11,454 (22.3)
Regional biopsy or aspiration	3832 (4.9)	2344 (4.6)
Sentinel	137 (0.2)	116 (0.2)
Others	1526 (1.9)	1032 (2.0)
Stage		
Stage IB	13,793 (17.6)	10,509 (20.4)
Stage II	6744 (8.6)	4399 (8.6)
Stage III	26,805 (34.2)	16,669 (32.4)
Stage IV	31,118 (39.7)	19,827 (38.6)
Chemotherapy		
No	41,196 (52.5)	28,452 (55.3)
Yes	37,264 (47.5)	22,952 (44.7)
Bone metastasis		
No	34,179 (43.6)	23,498 (45.7)
Yes	5716 (7.3)	3095 (6.0)
Others	38,565 (49.2)	24,811 (48.3)
Brain metastasis		
No	36,982 (47.1)	24,583 (47.8)
Yes	2831 (3.6)	1974 (3.8)
Others	38,647 (49.3)	24,847 (48.3)
Liver metastasis		
No	36,765 (46.9)	24,953 (48.5)
Yes	3067 (3.9)	1639 (3.2)
Others	38,628 (49.2)	24,812 (48.3)
Lung metastasis		
No	34,414 (43.9)	23,054 (44.8)
Yes	5309 (6.8)	3458 (6.7)
Others	38,737 (49.4)	24,892 (48.4)
First malignant indicator		
No	18,040 (23.0)	11,713 (22.8)
Yes	60,420 (77.0)	39,691 (77.2)
Age at diagnosis (year)		
<45	1096 (1.4)	900 (1.8)
≥45, <55	5622 (7.2)	3657 (7.1)
≥55, <65	17,933 (22.9)	10,178 (19.8)
≥65, <75	27,572 (35.1)	18,088(35.2)
≥75	26,237 (33.4)	18,581 (36.1)
Insurance status		
Medicaid	7935 (10.1)	5502 (10.7)
Insured or no specifics	49,351 (62.9)	32,578 (63.4)
Uninsured	1642 (2.1)	852 (1.7)
Blanks or unknown	19,532 (24.9)	12,472 (24.3)
Marital status		
Married or domestic partner	47,556 (60.6)	19,759 (38.4)
Divorced or separated or single or windowed	28,203 (35.9)	29,526 (57.4)
Unknown	2701 (3.4)	2119 (4.1)
High school education (Score)		
≤1000	15,562 (19.8)	10,782 (21.0)
1000–2000	40,366 (51.4)	27,093 (52.7)
2000–3000	19,808 (25.2)	12,122 (23.6)
≥3000	2724 (3.5)	1407 (2.7)
Median family income ($/month)		
≤5000	11,248 (14.3)	5820 (11.3)
5000–7000	37,995 (48.4)	24,593 (47.8)
7000–9000	20,258 (25.8)	14,123 (27.5)
>9000	8959 (11.4)	6868 (13.4)

SCC, squamous-cell carcinoma; AD, adenocarcinoma; LCC, large-cell lung carcinoma.

**Table 2:** Overall survival outcomes in NSCLC patients by sex across clinical stages IB–IV.

Stage	Sex	Mean Survival, Months (95% CI)	Median Survival, Months (95% CI)
Stage IB	Male	52.82 (51.85–53.80)	33.00 (31.88–34.12)
Stage IB	Female	66.29 (65.06–67.52)	52.00 (49.53–54.47)
Stage II	Male	44.19 (42.88–45.50)	23.00 (21.93–24.07)
Stage II	Female	52.87 (51.09–54.66)	30.00 (27.95–32.05)
Stage III	Male	25.09 (24.60–25.59)	11.00 (10.77–11.23)
Stage III	Female	31.35 (30.62–32.09)	13.00 (12.63–13.37)
Stage IV	Male	10.78 (10.51–11.04)	4.00 (3.90–4.10)
Stage IV	Female	15.14 (14.71–15.57)	5.00 (4.85–5.15)

Abbreviations: NSCLC, non-small-cell lung cancer; OS, overall survival; CI, confidence interval.

**Table 3:** Overall survival according to chemotherapy status, stratified by sex and stage in patients with NSCLC.

Stage	Chemotherapy	Sex	Mean OS (Months)	95% CI	Median OS (Months)	95% CI
Stage IB	No	Male	51.52	50.42–52.62	32.00	30.74–33.26
		Female	65.42	64.04–66.80	52.00	49.30–54.70
	Yes	Male	56.85	54.78–58.92	35.00	32.51–37.49
		Female	69.42	66.71–72.14	52.00	45.62–58.38
Stage II	No	Male	35.95	34.04–37.35	16.00	14.87–17.13
		Female	41.18	38.96–43.40	19.00	17.34–20.66
	Yes	Male	52.92	50.93–54.91	30.00	27.95–32.05
		Female	64.78	62.08–67.49	46.00	42.01–49.99
Stage III	No	Male	16.56	15.95–17.18	5.00	4.78–5.22
		Female	23.65	22.67–24.63	7.00	6.61–7.39
	Yes	Male	31.21	30.49–31.92	15.00	14.66–15.34
		Female	37.72	36.65–38.78	19.00	18.38–19.62
Stage IV	No	Male	7.20	6.88–7.52	2.00	1.96–2.05
		Female	10.99	10.45–11.52	2.00	1.90–2.10
	Yes	Male	14.59	14.17–15.01	8.00	7.84–8.16
		Female	19.65	18.98–20.33	10.00	9.73–10.28

**Table 4:** Multivariable Cox proportional hazards models for overall survival stratified by clinical stage.

Covariate	Stage IB HR	*p* Value	Stage II HR	*p* Value	Stage III HR	*p* Value	Stage IV HR	*p* Value
Sex (female vs. male)	0.766 (0.737–0.793)	<0.001	0.797 (0.757–0.836)	<0.001	0.846 (0.827–0.866)	<0.001	0.857 (0.840–0.874)	<0.001
Race: Black vs. White	0.981 (0.923–1.035)	0.505	1.002 (0.927–1.084)	0.967	0.973 (0.942–1.006)	0.096	0.975 (0.947–1.001)	0.068
Race: Others vs. White	0.872 (0.803–0.950)	0.001	0.919 (0.815–1.034)	0.163	0.870 (0.827–0.916)	<0.001	0.876 (0.842–0.916)	<0.001
Region: North vs. East	0.986 (0.929–1.047)	0.633	1.029 (0.949–1.120)	0.495	0.952 (0.916–0.988)	<0.001	0.875 (0.871–0.936)	<0.001
Region: Southwest vs. East	1.054 (0.954–1.167)	0.306	1.069 (0.919–1.242)	0.387	0.950 (0.888–1.017)	0.134	0.984 (0.928–1.043)	0.591
Region: Northwest vs. East	0.953 (0.924–1.004)	0.024	1.058 (1.000–1.122)	0.055	0.996 (0.970–1.023)	0.736	0.993 (0.970–1.016)	0.549
Primary tumor site: Middle vs. Upper lobe	0.960 (0.874–1.033)	0.337	0.943 (0.846–1.078)	0.348	1.090 (1.029–1.155)	0.004	0.957 (0.910–1.007)	0.087
Primary tumor site: Lower vs. Upper lobe	1.088 (1.053–1.131)	<0.001	0.999 (0.949–1.053)	0.984	1.125 (1.097–1.154)	<0.001	1.029 (1.006–1.053)	0.013
Primary tumor site: NOS vs. Upper lobe	1.423 (1.256–1.616)	<0.001	1.063 (0.936–1.250)	0.416	1.416 (1.357–1.478)	<0.001	1.105 (1.077–1.136)	<0.001
Primary tumor site: Overlapping lesion vs. Upper lobe	1.102 (0.938–1.275)	0.213	1.098 (0.937–1.303)	0.266	1.310 (1.198–1.434)	<0.001	1.250 (1.156–1.353)	<0.001
Primary tumor site: Main bronchus vs. Upper lobe	1.623 (1.461–1.779)	<0.001	0.956 (0.855–1.090)	0.471	1.271 (1.220–1.324)	<0.001	1.226 (1.178–1.275)	<0.001
Tumor grade: Grade II vs. I	1.089 (1.012–1.163)	0.017	0.935 (0.822–1.054)	0.287	1.217 (1.143–1.297)	<0.001	1.191 (1.126–1.259)	<0.001
Tumor grade: Grade III vs. I	1.098 (1.020–1.180)	0.012	0.983 (0.865–1.111)	0.793	1.256 (1.179–1.338)	<0.001	1.343 (1.271–1.419)	<0.001
Tumor grade: Grade IV vs. I	1.073 (0.943–1.236)	0.306	0.952 (0.780–1.142)	0.612	1.380 (1.253–1.519)	<0.001	1.477 (1.368–1.595)	<0.001
Tumor grade: Unknown vs. I	1.387 (1.299–1.506)	<0.001	1.043 (0.913–1.184)	0.528	1.278 (1.201–1.361)	<0.001	1.369 (1.298–1.443)	<0.001
Histology: AD vs. SCC	0.614 (0.583–0.642)	<0.001	0.771 (0.719–0.832)	<0.001	0.703 (0.677–0.731)	<0.001	0.737 (0.717–0.759)	<0.001
Histology: LCC vs. SCC	0.931 (0.847–1.002)	0.095	1.069 (0.962–1.192)	0.218	1.075 (1.028–1.125)	0.002	1.046 (1.013–1.080)	0.006
Chemotherapy (Yes vs. No)	0.889 (0.837–0.910)	<0.001	0.642 (0.611–0.673)	<0.001	0.562 (0.550–0.575)	<0.001	0.510 (0.500–0.520)	<0.001
Lymph nodes removed: ≥4 vs. 0–3	0.481 (0.456–0.492)	<0.001	0.469 (0.444–0.493)	<0.001	0.443 (0.428–0.459)	<0.001	0.371 (0.350–0.393)	<0.001
Lymph nodes removed: Regional biopsy/aspiration vs. 0–3	0.758 (0.646–0.889)	0.001	0.791 (0.665–0.945)	0.009	0.904 (0.864–0.945)	<0.001	0.956 (0.915–1.000)	0.048
Lymph nodes removed: Sentinel lymph node biopsy vs. 0–3	0.532 (0.396–0.727)	<0.001	0.445 (0.305–0.644)	<0.001	0.380 (0.286–0.504)	0.001	0.499 (0.306–0.816)	0.006
Lymph nodes removed: Others vs. 0–3	0.555 (0.494–0.606)	<0.001	0.542 (0.471–0.625)	<0.001	0.645 (0.595–0.698)	<0.001	0.748 (0.693–0.808)	<0.001
Bone metastasis (Yes vs. No)	-	-	-	-	1.854 (1.287–2.586)	<0.001	1.483 (1.443–1.526)	<0.001
Brain metastasis (Yes vs. No)	-	-	-	-	2.282 (1.257–3.798)	<0.001	1.376 (1.252–1.608)	<0.001
Liver metastasis (Yes vs. No)	-	-	-	-	-	-	1.473 (1.419–1.529)	<0.001
Lung metastasis (Yes vs. No)	-	-	-	-	1.491 (1.314–1.687)	<0.001	1.079 (1.060–1.105)	<0.001
First malignant indicator (Yes vs. No)	0.943 (0.899–0.968)	0.002	0.937 (0.891–0.991)	0.016	1.050 (1.023–1.077)	<0.001	1.147 (1.120–1.174)	<0.001
Diagnosis Age: 45–54 vs. <45	1.360 (1.068–1.743)	0.014	1.282 (0.980–1.716)	0.082	1.066 (0.961–1.184)	0.231	1.119 (1.040–1.203)	0.003
Diagnosis Age: 55–64 vs. <45	1.679 (1.338–2.124)	<0.001	1.486 (1.147–1.962)	0.004	1.115 (1.010–1.232)	0.031	1.158 (1.081–1.239)	<0.001
Diagnosis Age: 65–74 vs. <45	2.044 (1.633–2.585)	<0.001	1.806 (1.394–2.379)	<0.001	1.259 (1.141–1.390)	<0.001	1.206 (1.127–1.395)	<0.001
Diagnosis Age: ≥75 vs. <45	2.812 (2.248–3.560)	<0.001	2.331 (1.792–3.066)	<0.001	1.474 (1.336–1.629)	<0.001	1.303 (1.217–1.395)	<0.001
Insurance: Insured vs. Medicaid	0.761 (0.731–0.830)	<0.001	0.836 (0.763–0.906)	<0.001	0.900 (0.868–0.935)	<0.001	0.945 (0.915–0.974)	<0.001
Insurance: Uninsured vs. Medicaid	0.952 (0.784–1.132)	0.598	1.125 (0.906–1.408)	0.295	1.094 (1.006–1.192)	0.038	1.043 (0.976–1.112)	0.209
Insurance: Unknown vs. Medicaid	0.806 (0.765–0.881)	<0.001	0.873 (0.789–0.955)	0.005	0.954 (0.915–0.996)	0.029	0.973 (0.938–1.009)	0.148
Marital status: Not married vs. Married	1.143 (1.115–1.199)	<0.001	1.158 (1.099–1.213)	<0.001	1.107 (1.082–1.133)	<0.001	1.066 (1.045–1.088)	<0.001
Marital status: Unknown vs. Married	1.062 (0.972–1.179)	0.218	1.020 (0.891–1.153)	0.761	1.056 (0.995–1.121)	0.072	1.013 (0.965–1.065)	0.595
Education Score: 1000–2000 vs. ≤1000	0.965 (0.916–1.010)	0.159	1.033 (0.966–1.104)	0.335	0.998 (0.967–1.030)	0.888	0.959 (0.934–0.986)	0.002
Education Score: 2000–3000 vs. ≤1000	1.046 (0.973–1.117)	0.199	0.980 (0.891–1.076)	0.680	0.995 (0.952–1.040)	0.819	0.964 (0.932–1.006)	0.036
Education Score: ≥3000 vs. ≤1000	1.084 (0.952–1.210)	0.187	1.023 (0.866–1.208)	0.793	1.022 (0.948–1.103)	0.573	0.976 (0.917–1.047)	0.453
Family Income ($/month): 5000–7000 vs. ≤5000	0.954 (0.901–1.022)	0.141	0.982 (0.872–1.038)	0.611	0.994 (0.961–1.038)	0.739	0.975 (0.944–1.012)	0.126
Family Income ($/month): 7000–9000 vs. ≤5000	0.865 (0.808–0.942)	<0.001	0.925 (0.801–0.990)	0.046	0.949 (0.906–0.998)	0.006	0.938 (0.903–0.983)	0.001
Family Income ($/month): >9000 vs. ≤5000	0.804 (0.744–0.886)	<0.001	0.884 (0.769–0.976)	0.008	0.902 (0.855–0.955)	<0.001	0.903 (0.864–0.953)	<0.001

## Data Availability

The data presented in this study are openly available in Surveillance, Epidemiology, and End Results (SEER) Program at https://seer.cancer.gov/ (accessed on 26 May 2025).

## Supplementary Materials

The following supporting information can be downloaded at: https://www.mdpi.com/article/10.3390/healthcare14070966/s1, Figure S1: Comparison of overall survival between male and female cohorts stratified by age in stage IB patients with NSCLC; Figure S2: Comparison of overall survival between male and female cohorts stratified by age in stage II patients with NSCLC; Figure S3: Comparison of overall survival between male and female cohorts stratified by age in stage III patients with NSCLC; Figure S4: Comparison of overall survival between male and female cohorts stratified by age in stage IV patients with NSCLC. Table S1. Median Follow-up Duration of NSCLC Patients Stratified by Sex at Stages IB through IV; Table S2. Overall survival according to chemotherapy status, stratified by sex and calendar period in patients with NSCLC.
