# Supplementary material for: Sex Differences in Overall Survival Among Patients with Non-Small-Cell Lung Cancer Across Clinical Stages: A Population-Based SEER Study
Source: Healthcare (Basel). 2026 Apr 7;14(7):966. doi: 10.3390/healthcare14070966 (PMC13074028; doi:10.3390/healthcare14070966)
Supplement: Supplementary file 1 [file healthcare-14-00966-s001.zip › healthcare-4182235-supplementary.pdf]

Supplement materials

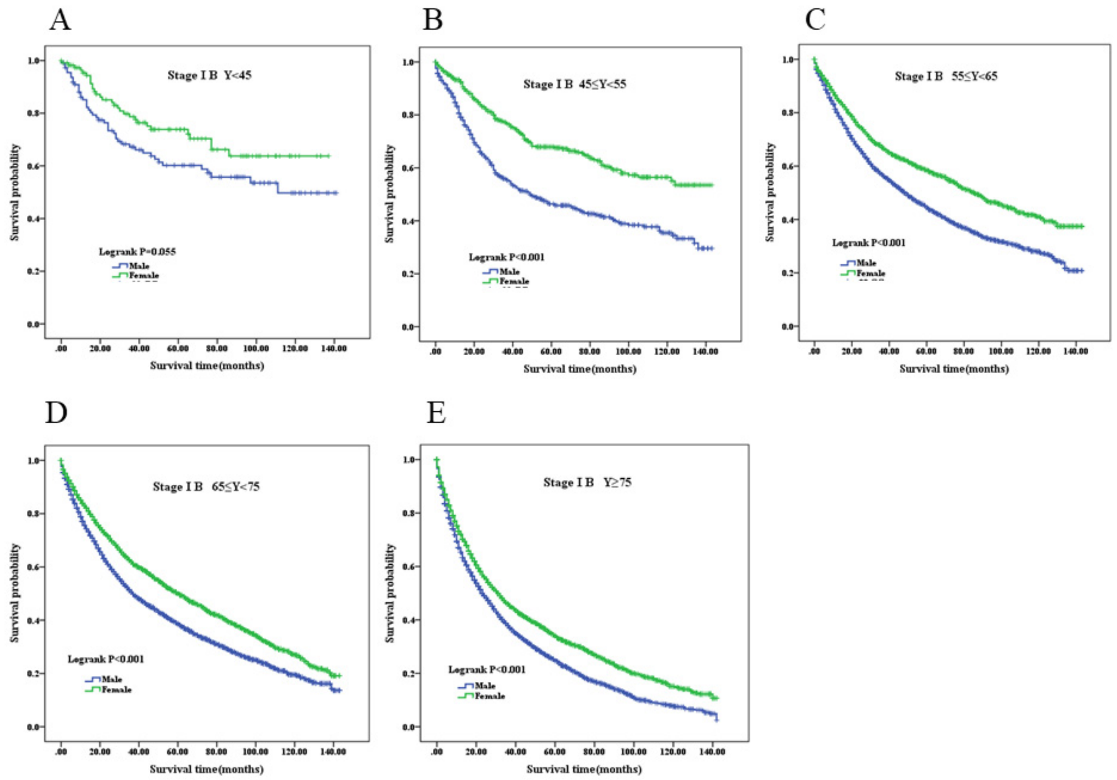

Figure S1. Comparison of overall survival between male and female cohorts stratified by age in stage IB patients with NSCLC. **A**. Stage IB (age < 45y); **B**. Stage IB (45y ≤ age < 55y); **C**. Stage IB (55y ≤ age < 65y); **D**. Stage IB (65y ≤ age < 75y). **E**. Stage IB (age ≥ 75y). NSCLC, non–small cell lung cancer.

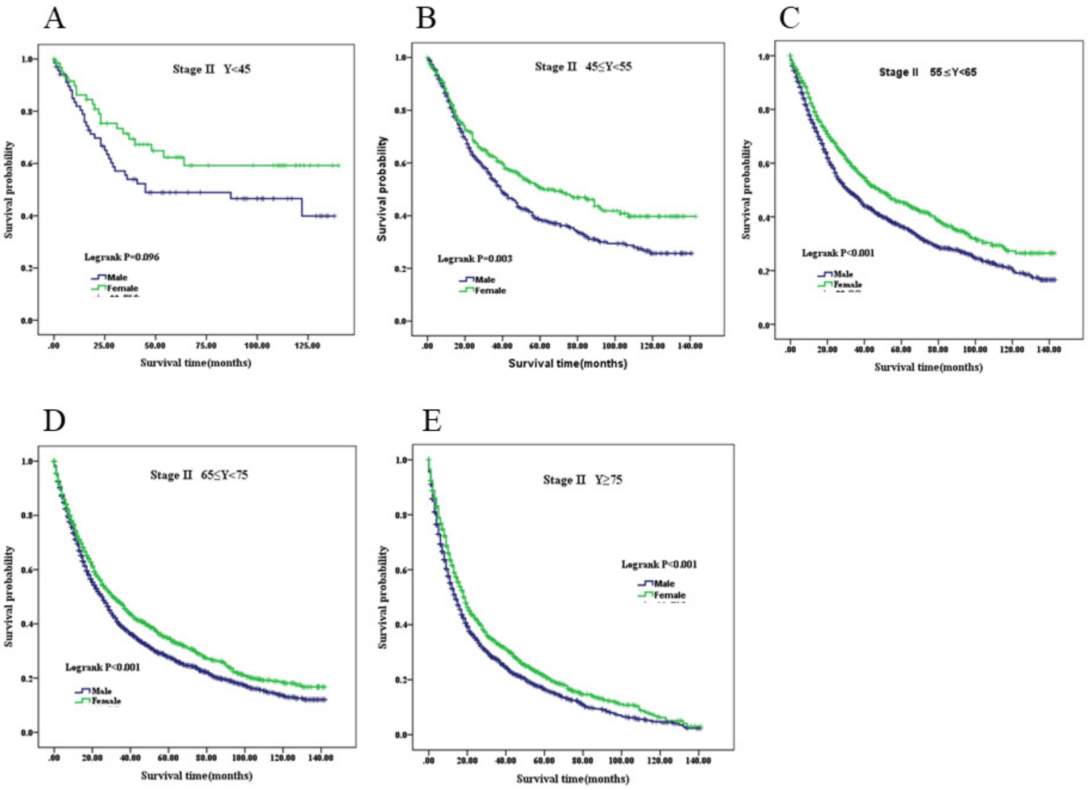

Figure S2. Comparison of overall survival between male and female cohorts stratified by age in stage II patients with NSCLC. **A**. Stage II (age < 45y); **B**. Stage II (45y ≤ age < 55y); **C**. Stage II (55y ≤ age < 65y); **D**. Stage II (65y ≤ age < 75y). **E**. Stage II (age ≥ 75y). NSCLC, non–small cell lung cancer.

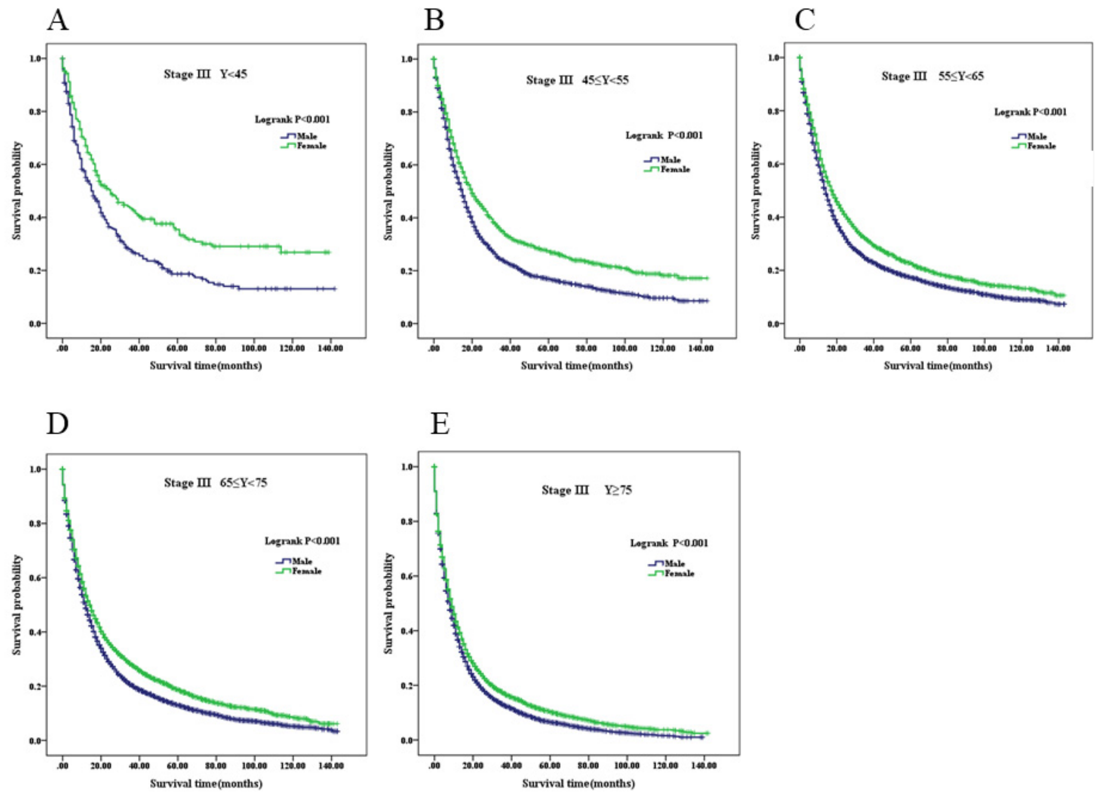

Figure S3. Comparison of overall survival between male and female cohorts stratified by age in stage III patients with NSCLC. **A**. Stage III (age < 45y); **B**. Stage III (45y ≤ age < 55y); **C**. Stage III (55y ≤ age < 65y); **D**. Stage III (65y ≤ age < 75y). **E**. Stage III (age ≥ 75y). NSCLC, non–small cell lung cancer.

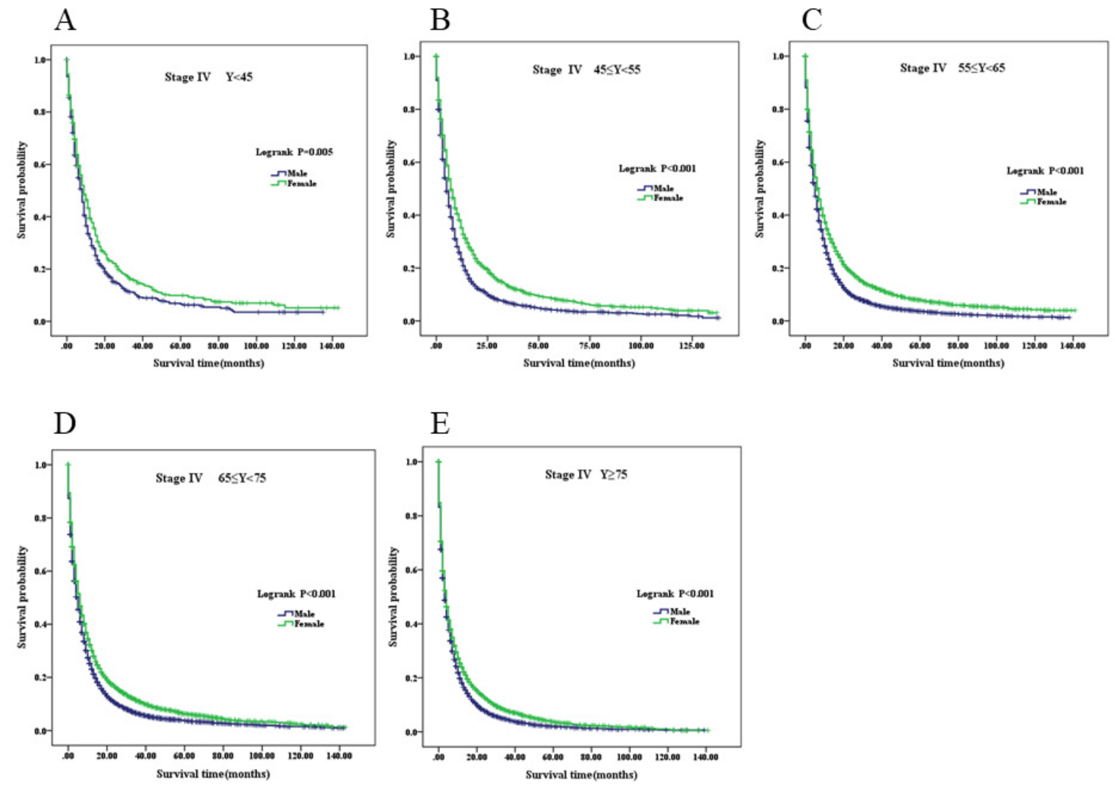

Figure S4. Comparison of overall survival between male and female cohorts stratified by age in stage IV patients with NSCLC. **A**. Stage IV (age < 45y); **B**. Stage IV (45y ≤ age < 55y); **C**. Stage IV (55y ≤ age < 65y); **D**. Stage IV (65y ≤ age < 75y). **E**. Stage IV (age ≥ 75y). NSCLC, non–small cell lung cancer.

Table S1. Median Follow-up Duration of NSCLC Patients Stratified by Sex at Stages IB through IV.

Stage	Chemotherapy	Mean follow-up (months)	95% CI	Median follow-up (months)	95% CI
**Stage IB**					
Male	No	69.22	68.06-70.37	69.00	66.95-71.02
	Yes	70.69	68.64-72.73	69.00	65.08-72.92
Female	No	66.53	65.39-67.67	65.00	63.15-66.85
	Yes	69.28	66.03-71.53	68.00	63.98-72.02
**Stage II**					
Male	No	75.67	73.02-78.32	77.00	71.86-82.14
	Yes	68.14	66.12-70.17	69.00	65.02-72.98
Female	No	73.02	70.11-75.94	74.00	67.45-80.55
	Yes	64.65	62.41-66.89	61.00	56.97-65.03
**Stage III**					
Male	No	71.30	69.06-73.53	71.00	66.58-75.42
	Yes	66.14	64.87-67.40	63.00	60.82-65.18
Female	No	69.04	66.93-71.15	66.00	62.93-69.07
	Yes	64.85	63.41-66.30	63.00	60.46-65.55
**Stage IV**					
Male	No	67.20	64.09-70.31	66.00	61.18-70.82
	Yes	64.37	61.94-66.81	60.00	56.18-63.82
Female	No	68.35	65.43-71.27	66.00	61.92-70.08
	Yes	66.49	64.24-68.75	67.00	62.85-71.15

Table S2. Overall survival according to chemotherapy status, stratified by sex and calendar period in patients with NSCLC.

	Sex	Chemotherapy	Mean OS (months	95% CI	Median OS (months)	95% CI
2004-2007	Male	No	22.96	22.32-23.60	6.00	5.74-6.26
		Yes	27.92	27.21-28.62	12.00	11.67-12.33
	Female	No	30.85	29.92-31.78	8.00	7.52-8.48
		Yes	34.67	33.64-35.70	15.00	14.39-15.61
2008-2011	Male	No	20.51	20.01-21.01	6.00	5.72-6.28
		Yes	25.14	24.63-25.65	13.00	12.65-13.35
	Female	No	28.26	27.55-28.97	10.00	9.43-10.57
		Yes	30.43	29.70-31.16	16.00	15.42-16.58
2012-2015	Male	No	14.76	14.43-15.09	6.00	5.07-6.29
		Yes	19.67	19.32-20.00	13.00	12.62-13.38
	Female	No	18.43	18.01-18.85	9.00	8.41-9.59
		Yes	22.81	22.37-23.26	17.00	16.27-17.73
